# Atrial flutter-related health care use and costs: An analysis of a nationally representative administrative claims database in the United States

**DOI:** 10.1016/j.hroo.2023.04.003

**Published:** 2023-04-24

**Authors:** Abhishek Deshmukh, Maximiliano Iglesias, Rahul Khanna, Tara Beaulieu

**Affiliations:** ∗ Division of Cardiovascular Disease, Mayo Clinic, Rochester, Minnesota; † Johnson & Johnson Medical Devices, Franchise Health Economics and Market Access, Irvine, California; ‡ MedTech Epidemiology and Real-World Data Sciences, Johnson and Johnson, New Brunswick, New Jersey

**Keywords:** Arrhythmia, Atrial flutter, Cost burden, Health care use burden, United States

## Abstract

**Background:**

Atrial flutter (AFL) is a common arrhythmia associated with significant morbidity, yet the incremental burden of this condition has not been well documented.

**Objective:**

Using real-world data, we sought to evaluate the healthcare use and cost burden of incident AFL in the United States

**Methods:**

From 2017 to 2020, persons with an incident diagnosis of AFL were identified through Optum Clinformatics, a nationally representative administrative claims database of commercially insured individuals in the United States. We constructed 2 cohorts (AFL patient; non-AFL comparator) and used a matching weights method to balance covariates between cohorts. Using logistic regression and general linear models, 12-month all-cause and cardiovascular (CV)-related health care use (inpatient, outpatient, emergency room [ER] visits, and other) as well as medical expenditures were compared between the matched cohorts.

**Results:**

The matching weight sample sizes were 13,270 for AFL and 13,683 for the non-AFL cohorts. In the AFL cohort, ∼71% were at least 70 years of age, 62% identified as male, and 78% identified as White. The AFL cohort had significantly higher health care use, including all-cause (relative risk [RR] 1.14; 95% confidence interval [CI] 1.11–1.18) and CV-related ER visits (RR 1.60; 95% CI 1.52–1.70) compared with the non-AFL cohort. Mean total health care costs (per patient annually) were almost $21,783 (95% CI $18,967–$24,599) higher among patients with AFL compared to those without AFL ($71,201 vs $49,418, respectively; *P* <.001).

**Conclusion:**

Amidst the backdrop of an aging population, findings from this study draw attention to the importance of timely and adequate treatment of AFL.

## Graphical abstract

**Figure:**
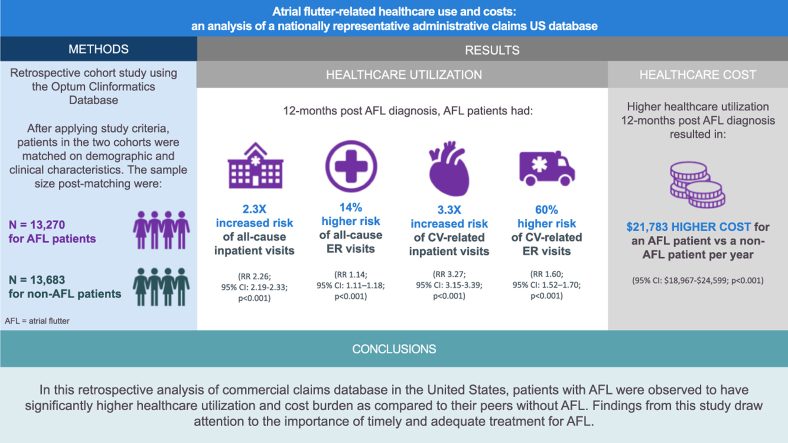

**Key Findings**

▪ The incremental burden of incident atrial flutter (AFL) has not been previously examined.
▪ In our study of 13,270 patients with AFL and 13,683 patients without AFL, patients with AFL were observed to have significantly higher health care use (including all-cause and cardiovascular-related use) as well as medical expenditure burden compared to patients without AFL.
▪ Our findings suggest considerable patient and public health burden associated with AFL.

## Introduction

Atrial flutter (AFL) is a relatively common type of arrhythmia,^1^, ^2^, ^3^ with a previously reported incidence rate of 88 per 100,000 person-years among a general population in the United States (US) (specifically Marshfield Epidemiologic Study Area).^2^^,^^3^ As with atrial fibrillation [AF]),^4^, ^5^, ^6^ studies have suggested that AFL incidence rates are likely to increase.^1^ By 2050, the number of individuals with AFL alone is anticipated to be 150,000, with another 440,000 having concurrent AFL and AF in the US.^1^ The anticipated rise in AFL incidence and prevalence rates (taken with the relative commonality of AFL, to begin with) is likely to place a considerable burden on patients and society, because AFL can lead to substantial morbidity, including heart muscle damage, stroke,^7^ and diminished quality of life.^8^

This study aimed to examine the incremental health care use and cost burden associated with an incident diagnosis of AFL. Although a few studies have examined the health care burden of patients with both AF and AFL,^9^, ^10^, ^11^ to date no study has depicted the incremental health care burden associated with AFL as a standalone disease. Furthermore, there is limited understanding of AFL burden by sex categorization.

## Methods

### Data source

Data for this study were derived from the Optum Clinformatics database.^12^ The Optum database comprises insurance claims data for UnitedHealthcare plan members in the US, and includes medical services, prescription fill, demographic, and enrollment information for those individuals. The New England Institutional Review Board (IRB) determined that studies using data derived from the Optum database are exempt from study-specific IRB review as human subjects are not actively participating or involved in these studies, and all data used in this analysis were deidentified.^13^^,^^14^ Given the observational nature of the study using a de-identified retrospective database, informed consent was not required.

### Study population

Individuals were included in the AFL cohort if they were at least 19 years of age and had at least 2 medical service visits with a primary diagnosis of AFL (based on *International Classification of Diseases, Tenth Revision, Clinical Modification* [ICD-10-CM] codes I48.92, I48.3, I48.4) within 90 days of each other in any setting between January 1, 2017, and March 31, 2020, with the first medical services visit considered as the index visit (incident diagnosis). Individuals were excluded if they had a record of a medical service visit with a diagnosis of AFL (primary or secondary) or an antiarrhythmic (AAD) drug prescription claim (including for amiodarone, disopyramide, dofetilide, dronedarone, flecainide, quinidine, propafenone, or sotalol) during the 12-month preindex period. Having multiple diagnoses of AFL allowed us to restrict our sample to actual AFL cases and excluded those with a false-positive diagnosis.

To better understand the incremental burden imposed by AFL, we identified a comparator cohort of individuals without AFL diagnosis. A pseudo-index date was provided for the comparator cohort based on the index date for the AFL cohort. Individuals in both cohorts were required to have continuous enrollment during the 12-month pre- and postindex dates.

As sensitivity analysis, we also broadened our inclusion criteria to at least 1 primary diagnosis of AFL (all other criteria remaining the same) and reran the analysis. The rationale for doing so and the findings from this sensitivity analysis are detailed in the Study limitations.

### Study variables

The primary outcomes of interest were all-cause, cardiovascular (CV)-related, and AFL-related inpatient, outpatient (eg, walk-in retail health clinic, ambulatory surgery center), emergency room (ER), and other medical services visits (eg, ambulance, mobile unit). The costs associated with medical visits, prescription, and total health care costs (sum of medical services and prescription costs) also were reported. All costs were reported in US$ and adjusted for medical inflation (and reported in 2021 US$). We also examined treatment provided to patients with AFL within 12 months of diagnosis. Treatment assessed included cardioversion, catheter ablation, and AAD use.

Covariates include age, sex (male, female), race/ethnic affiliation (White, Black, Asian, Hispanic, Unknown), geographic region (Northeast, Midwest, South, West), Elixhauser comorbidity score,^15^ CHA₂DS₂-VASc scores,^16^ obstructive sleep apnea, hyperthyroidism, history of cardiac surgery, and AF were assessed among the AFL and non-AFL cohorts using all available preindex visit date data.

### Statistical analysis

To achieve optimal balance of all study covariates (eg, age, geographic region, AF) between the AFL and non-AFL cohorts, the matching weight method of Yoshida et al^17^ was used. Standardized mean differences (SMDs) were used to assess the postmatch balance of covariates (SMD cutoff 0.25 or ≤–0.25 were considered for imbalance), and all remaining analyses were performed as weighted analyses. Logistic regression models were used to assess services, and general linear models were used to determine cost differences among the matched AFL and non-AFL cohorts. We performed subanalysis stratifying the cohorts by sex. For this analysis, separate cohorts of males and females were constructed for both AFL and non-AFL patients, respectively, and then matching weight methods were used (ie, female patients with AFL were matched to female patients without AFL; male patients with AFL were matched with male patients without AFL). We also performed subanalysis by individual AFL type cohorts (ie, atypical, typical, and unspecified AFL). Patients with record of multiple AFL types (eg, atypical and typical AFL) were classified as “unspecified AFL.” Each AFL type cohort was then matched to a different random subset of the non-AFL cohort.

Given the coexisting relationship of AFL and AF, we performed subanalysis when patients with AFL with concomitant diagnosis of AF were excluded (thus only focusing on patients with AFL without concomitant AF). This allowed us to better assess the true burden of AFL without AF. Outcome analyses were rerun to examine the incremental burden of AFL in these revised cohorts. Study analyses were conducted using R for Windows, Version 4.0.2.^18^

## Results

As depicted in the attrition table (Table 1), a final sample of 13,319 patients was identified in the AFL cohort based on study criteria. Approximately 16 million patients met the inclusion criteria for the non-AFL cohort. For the feasibility of analysis, we extracted a random sample of 6% of the non-AFL cohort, which resulted in 962,109 patients. After application of the matching weights procedure, the cohort sizes were 13,270 AFL and 13,683 non-AFL patients. The AFL and non-AFL matched cohorts were well balanced on all covariates (SMD ≥0.25 or ≤–0.25) (Table 2). Both cohorts were 62%-63% male, with ≈78% being White. Among patients in the AFL cohort, 19.8% had received cardioversion, 17.6% had undergone catheter ablation, and 25.5% had an AAD prescription claim in the 1-year period after diagnosis with AFL.

**Table 1:** Study cohort attrition for patients with AFL

Step	Criteria	N
1	Patient sample after including those who had 2 primary AFL diagnosis within a 90-day period between January 1, 2017, and March 31, 2020	60,905
2	Patient sample after including those age ≥19 years (age assessed at first service visit)	60,844
3	Patient sample after including those continuously enrolled in the 12-month preindex period and 12-month postindex periods	35,648
4	Patient sample after excluding those who had a medical services visit (any setting) with a primary or secondary diagnosis of AFL in the preindex period (all available data)	15,824
5	Patient sample after excluding those who had a prescription claim for an antiarrhythmic drug in the preindex period (all available data)	13,333
6	Patient sample after excluding those with an “Unknown” gender identity response, as well as those with “Unknown” and multiple geographic region responses	13,319
	AFL cohort	13,319

AFL = atrial flutter.

**Table 2:** Sample characteristics for patients with and without incident AFL

	Prematch			Postmatch		
	AFL (N = 13,319)	No AFL (N = 955,888)	SMD	AFL (N = 13,270)	No AFL (N = 13,683)	SMD
Age (categorical) (y)			1.254			0.039
19–39	87 (0.7)	252,577 (26.4)		87.0 (0.7)	86.4 (0.6)	
40–49	278 (2.1)	130,623 (13.7)		278.0 (2.1)	272.2 (2.0)	
50–59	899 (6.7)	144,683 (15.1)		899.0 (6.8)	860.5 (6.3)	
60–69	2615 (19.6)	173,893 (18.2)		2614.8 (19.7)	2538.4 (18.6)	
≥70	9440 (70.9)	254,112 (26.6)		9391.2 (70.8)	9925.0 (72.5)	
Male	8219 (61.7)	431,078 (45.1)	0.338	8170.0 (61.6)	8574.2 (62.7)	0.023
Geographic region			0.097			0.008
Northeast	1757 (13.2)	112,694 (11.8)		1757.0 (13.2)	1805.8 (13.2)	
Midwest	3332 (25.0)	233,489 (24.4)		3329.9 (25.1)	3453.0 (25.2)	
South	5170 (38.8)	413,803 (43.3)		5168.0 (38.9)	5284.7 (38.6)	
West	3060 (23.0)	195,902 (20.5)		3015.1 (22.7)	3139.1 (22.9)	
Race			0.291			0.013
Asian	233 (1.7)	41,719 (4.4)		233.0 (1.8)	234.4 (1.7)	
Black	1343 (10.1)	96,722 (10.1)		1342.1 (10.1)	1363.8 (10.0)	
Hispanic	871 (6.5)	110,512 (11.6)		870.8 (6.6)	863.6 (6.3)	
Unknown	542 (4.1)	67,954 (7.1)		542.0 (4.1)	554.7 (4.1)	
White	10,330 (77.6)	638,981 (66.8)		10,282.1 (77.5)	10,666.0 (78.0)	
Elixhauser score			1.230			0.032
0	0 (0.0)	201,207 (21.0)		0.0 (0.0)	0.0 (0.0)	
1–3	1945 (14.6)	409,395 (42.8)		1945.0 (14.7)	1851.0 (13.5)	
≥4	11,374 (85.4)	345,286 (36.1)		11,325.0 (85.3)	11,831.6 (86.5)	
CHADS_2_-VASc score			0.973			0.043
0	281 (2.1)	172,843 (18.1)		281.0 (2.1)	274.0 (2.0)	
1–3	4310 (32.4)	548,454 (57.4)		4285.0 (32.3)	4154.5 (30.4)	
≥4	8728 (65.5)	234,591 (24.5)		8703.9 (65.6)	9254.1 (67.6)	
Obstructive sleep apnea	3358 (25.2)	116,027 (12.1)	0.340	3333.9 (25.1)	3515.1 (25.7)	0.013
Hyperthyroidism	417 (3.1)	17,058 (1.8)	0.087	416.4 (3.1)	437.6 (3.2)	0.003
History of cardiac surgery	2215 (16.6)	25,476 (2.7)	0.487	2185.6 (16.5)	2383.4 (17.4)	0.025
Atrial fibrillation	8169 (61.3)	50,971 (5.3)	1.477	8120.0 (61.2)	8585.6 (62.7)	0.032
Year			1.385			0.008
2017	3792 (28.5)	113,048 (11.8)		3771.4 (28.4)	3894.8 (28.5)	
2018	4190 (31.5)	124,146 (13.0)		4166.4 (31.4)	4309.7 (31.5)	
2019	4433 (33.3)	139,409 (14.6)		4428.1 (33.4)	4573.4 (33.4)	
2020	904 (6.8)	579,285 (60.6)		904.0 (6.8)	904.6 (6.6)	

Values are given as n (%) unless otherwise indicated.

AFL = atrial flutter; SMD = standard mean difference.

Table 3 lists AFL-related health care use and costs for the AFL cohort in the 12-month postincident diagnosis. Almost 80% of the AFL cohort had AFL-related outpatient visits, 37% had inpatient visits, 6% had ER visits, and 12% had other medical visits. Mean cost per patient for outpatient visits was $7001 ± $20,109; for inpatient visits was $3845 ± $27,818; for ER visits was $291 ± $5014; and for other medical visits was $114 ± $1385. A comparison of health care use between the matched AFL and non-AFL patients is given in Table 4. All-cause health care use, including all-cause inpatient visits (relative risk [RR] 2.26; 95% confidence interval [CI] 2.19–2.33), outpatient visits (RR 1.01; 95% CI 1.01**–**1.02); ER visits (RR 1.14; 95% CI 1.11–1.18), and other medical visits (RR 1.11; 95% CI 1.08–1.13) was significantly higher among the AFL patients compared with the non-AFL patients. The AFL cohort also had substantially higher CV-related health care use compared to the non-AFL cohort, including CV-related inpatient visits (RR 3.27; 95% CI 3.15–3.39), outpatient visits (RR 2.86; 95% CI 2.82–2.89), ER visits (RR 1.60; 95% CI 1.52–1.70), and other medical visits (RR 1.40; 95% CI 1.35–1.45). Table 5 lists the average health care costs per patient in the 12-month postindex period among the AFL and non-AFL cohorts. Patients with AFL had $7640 higher mean all-cause inpatient costs (*P* <.001), $11,192 higher outpatient costs (*P* <.001), and $615 higher CV-related ER costs (*P* <.001) compared to patients without AFL. Average prescription drug costs were $1693 higher (*P* <0.001) and total health care costs were $21,783 higher (*P* <.001) for AFL patients compared to non-AFL patients.

**Table 3:** AFL-related health care utilization and average per patient costs in the 12-month postindex period

	AFL patients (N = 13,270)
AFL-related health care utilization	
Inpatient visits	4916.3 (37.0)
Outpatient visits	10,492.2 (79.1)
ER visits	815.1 (6.1)
Other medical visits∗	1560.5 (11.8)
AFL-related health care cost	
Inpatient visits	$3845 ± $27,818
Outpatient visits	$7001 ± $20,109
ER visits	$291 ± $5014
Other medical visits∗	$114 ± $1385

AFL = atrial flutter; ER = emergency room.

Values are given as n (%) or mean ± SD.

∗ Other medical costs included any medical visits that were not captured with the inpatient, outpatient, or ER visit categories (eg, pharmacy, ambulance, mobile unit, nursing facility, skilled nursing facility, or residential substance abuse treatment facility).

**Table 4:** Health care use among patients with AFL and patients without AFL in the 12-month postindex period

Outcome	AFL	No AFL	*P* value∗	RR (95% CI)
All-cause health care use				
Inpatient visits	59.1	35.2	<.001	2.26 (2.19–2.33)
Outpatient visits	99.3	97.4	<.001	1.01 (1.01–1.02)
ER visits	40.7	36.8	<.001	1.14 (1.11–1.18)
Other medical visits†	70.4	66.3	<.001	1.11 (1.08–1.13)
CV-related health care use				
Inpatient visits	52.6	23.5	<.001	3.27 (3.15–3.39)
Outpatient visits	97.6	70.5	<.001	2.86 (2.82–2.89)
ER visits	17.0	11.2	<.001	1.60 (1.52–1.70)
Other medical visits†	40.5	31.8	<.001	1.40 (1.35–1.45)

Values are given as % unless otherwise indicated.

AFL = atrial flutter; CI = confidence interval; CV = cardiovascular; ER = emergency room; RR = relative risk.

∗ From bivariate regression model.

† Other medical visits included any medical visits that were not captured with the inpatient, outpatient, or ER visit categories (eg, pharmacy, ambulance, residential substance abuse treatment facility).

**Table 5:** Health care costs among patients with AFL vs those without AFL in the 12-month postindex visit

	AFL	No AFL	Mean cost difference (95% CI)	*P* value∗
All-cause health care cost				
Inpatient visits	$33,024 ± $108,813	$25,384 ± $122,206	$7640 ($5206–$10,074)	<.001
Outpatient visits	$22,648 ± $37,086	$11,456 ± $26,029	$11,192 ($10,511–$11,873)	<.001
ER visits	$5827 ± $25,343	$4890 ± $16,665	$937 ($472–$1403)	<.001
Other medical visits†	$3745 ± $26,946	$3425 ± $23,269	$320 ($-208–$848)	.236
CV-related health care costs				
Inpatient visits	$15,129 ± $59,434	$6877 ± $79,127	$8252 ($6749–$9755)	<.001
Outpatient visits	$13,015 ± $28,429	$1818 ± $7561	$11,197 ($10,706–$11,687)	<.001
ER visits	$908 ± $8981	$293 ± $2343	$615 ($460–$770)	<.001
Other medical visits†	$757 ± $3246	$617 ± $3527	$140 ($73–$208)	<.001
Prescription costs	$5956 ± $13,894	$4263 ± $13,332	$1693 ($1422–$1966)	<.001
Total health care costs	$71,201 ± $13,0294	$49,418 ± $138,691	$21,783 ($18,967–$24,599)	<.001

Values are given as mean cost ± SD (in US$) unless otherwise indicated.

Abbreviations as in Table 4.

∗ From bivariate regression model.

† Other medical visits included any medical visits that were not captured with the inpatient, outpatient, or ER visit categories (eg, pharmacy, ambulance, residential substance abuse treatment facility).

**Figure 1:**
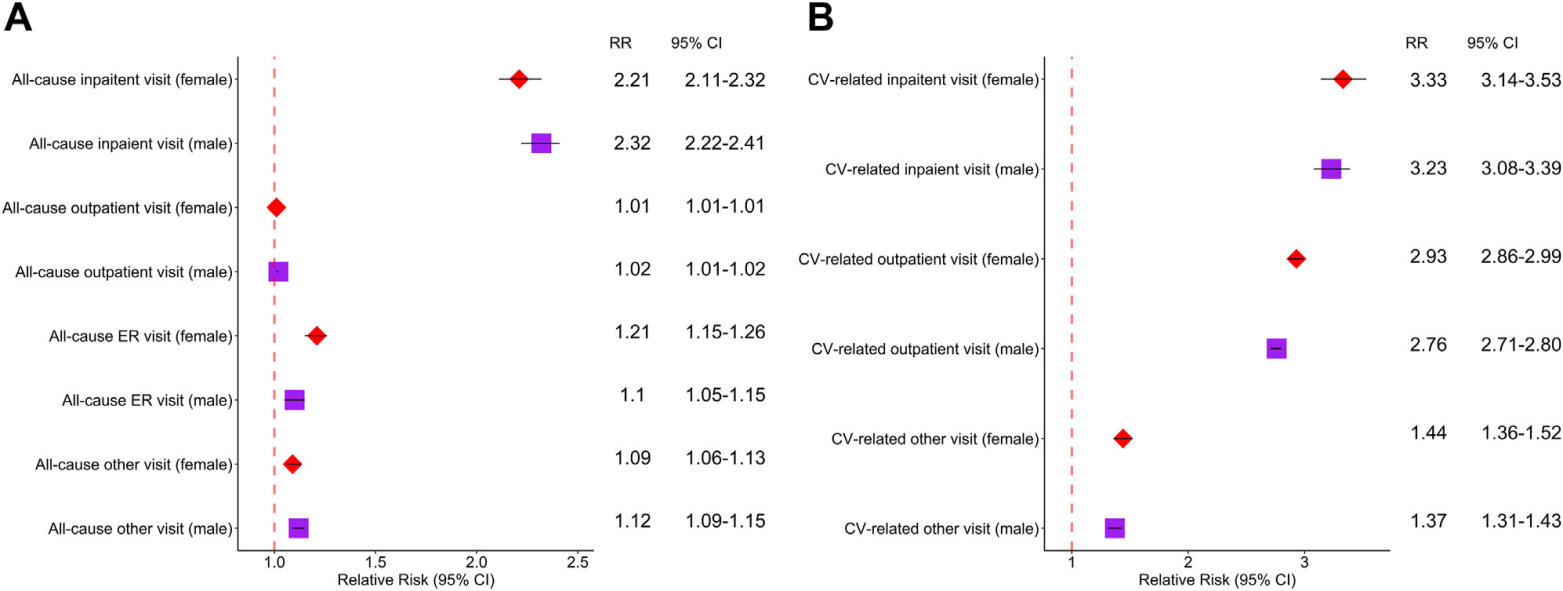
Relative risk of health care utilization by patients with atrial flutter (AFL) compared to those without AFL among males and females for all-cause visits **(A)** and cardiovascular (CV)-related visits **(B).** Findings are from bivariate regression models. Not all outcomes are reported as the following message was received while running certain models: “Fitted probabilities numerically 0 or 1 occurred,” indicative of the potential for separation or convergence issues.^19^ CI = confidence interval; ER = emergency room; RR = relative risk.

Results of the subanalysis by sex are shown in Figure 1. After stratifying AFL and non-AFL patients by sex (and performing a new matching among the subset patients), all-cause and CV-related health care utilization remained significantly higher among males in the AFL cohort compared to males in the non-AFL cohort, including all-cause inpatient visits (RR 2.32; 95% CI 2.22–2.41), CV-related inpatient visits (RR 3.23; 95% CI 3.08–3.39), and CV-related outpatient visits (RR 2.76; 95% CI 2.71–2.80). Similar results were observed among female-specific analyses.

For the subanalysis performed after the removal of patients with AF, the RRs of all-cause and CV-related health care use, including all-cause inpatient visits (RR 2.48; 95% CI 2.38–2.60) and CV-related outpatient visits (RR 3.30; 95% CI 3.25–3.34), were significantly higher for the AFL cohort compared to the non-AFL cohort (Supplemental Table 1). Total associated costs were $31,186 higher (*P* <.001) for the AFL cohort compared to the non-AFL cohort (Supplemental Table 2).

**Figure 2:**
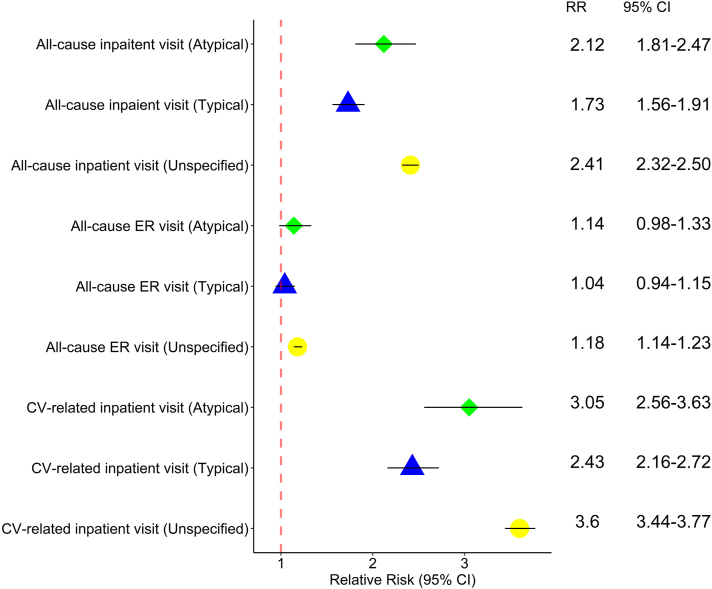
Relative risk of health care utilization by patients with atrial flutter (AFL) compared to those without AFL among patients with atypical, typical, and unspecified AFL for all-cause and cardiovascular-related visits. Abbreviations as in Figure 1.

Findings from the subanalysis by AFL type are shown in Figure 2. Patients with atypical AFL had a significantly higher risk of all-cause (RR 2.21; 95% CI 1.81–2.47) and CV-related inpatients visits (RR 3.05; 95% CI 2.56–3.63) compared to patients without AFL. Similar results were observed for patients with typical AFL and unspecified AFL.

## Discussion

Our study suggests that incident AFL imposes significant health care use and cost burden. To the best of our knowledge, this is the first study to examine the incremental burden of AFL. We found significantly higher all-cause and CV-related health care use among patients with an incident diagnosis of AFL compared to our matched comparison group (ie, those without AFL with comparable demographics and comorbidity status), including higher inpatient, outpatient, ER, and other medical visits. Health care costs, including all-cause, CV-related, prescription, and total health care costs, were significantly higher among patients with AFL compared to a non-AFL cohort with similar comorbidity status at baseline. Results were observed to be consistent across different analysis approaches, including by sex, AFL type, and removal of patients with concomitant AF.

Given the lack of previous such studies, it is difficult for us to put into perspective the results observed in our study. Our findings align with previous studies in the area of AF/AFL. For example, a US-based retrospective cohort study that examined health care use in patients with AF/AFL (plus at least 1 additional risk factor aside from heart failure) compared to matched non-AF/AFL patient controls found that all-cause hospitalizations were ≈3 times more likely. CV-related outpatient visits were ≈4 times as likely among those in the AF/AFL cohort compared to the non-AF/AFL control cohort in the 1-year postindex period (*P* <.0001).^20^ Overall mean health care cost was also significantly higher among the AF/AFL cohort compared to the control cohort (*P* <.0001).^20^ A recent US-based study examining the incremental health burden associated with incident AF reported the total health care cost burden of an AF cohort to be approximately $28,000 greater per year (per individual) compared with the non-AF cohort (*P* <.001).^21^ Although such studies are not directly comparable, the results from our study suggest that AFL, by itself and in the presence of AF, places a considerable health burden, including an economic burden on par with that of AF on patients, providers, and payers. Our study findings highlight the importance of ensuring timely and adequate treatment of AFL to minimize the burden on patients. Catheter ablation has increasingly become an important treatment modality for patients with AFL. AFL ablation is associated with a high rate of single-procedure efficacy of >95%^22^ and a low incidence of AFL recurrence.^23^ The American College of Cardiology/American Heart Association guidelines (2015) and the European Society of Cardiology guidelines (2019) indicate that catheter ablation of typical AFL often is preferred to long-term pharmacologic therapy.^24^^,^^25^ Notably, a US-based study of 33,004 individuals with an AFL diagnosis specifically found significant reductions in health care use, including inpatient hospitalizations (hazard ratio [HR] 0.88; 95% CI 0.84–0.92; *P* <.001), and ER visits (HR 0.60; 95% CI 0.54–0.65; *P* <.001) after catheter ablation compared to those with an AFL diagnosis who had not undergone an ablation.^26^ Given the considerable health care use and cost burden imposed by AFL as seen in this study, early catheter ablation among AFL patients could potentially help alleviate the burden.

When examining AFL burden by sex, we identified a considerable incremental burden of medical services use among females and males with AFL compared to females and males without AFL. Another potentially important finding was that the burden seemed to be slightly more pronounced among females across most all-cause and CV-related health care utilization categories. Further investigation is warranted to confirm and elucidate sex affiliation disparities in the burden of AFL.

Results from analysis by AFL type were consistent with overall main analysis wherein all AFL patients were combined. The incremental health care burden associated with AFL diagnosis was existent for atypical, typical, and unspecified AFL patients. These results suggest that although the underlying mechanisms differentiating typical and atypical AFL could vary, the overall adverse impact on patient health remains considerable across these AFL types.

### Study limitations

First, it is vital to consider that the Optum database primarily includes commercial patients (0–65 years old), and Medicare part C patients (>65 years of age). As such, our results may not be generalizable to those without commercial insurance or to individuals with Medicare fee-for-service insurance. Second, coding errors during the processing of claims may have occurred and could affect study results. Third, it is important to consider the accuracy and validity of the diagnostic codes that we used to define AFL. Although there is some evidence suggesting that the diagnostic accuracy of certain AF/AFL codes in certain administrative databases is high with sensitivity and specificity of 95%, positive predictive value of 97%, and negative predictive value of 92%,^27^ this remains an important area for future research. Fourth, we set one of our inclusion criteria to consider only patients with at least 2 AFL diagnoses within a 3-month window to mitigate the potential for inclusion of those with a false-positive diagnosis. It is important to consider that such an approach may result in a cohort that consists of patients with higher frequencies of health care utilization, which could bias our findings against the null hypothesis. As such, we reran our analysis with an AFL cohort consisting of patients with at least 1 AFL diagnosis. These patients with AFL were then matched to patients without AFL for the assessment of study outcomes. Results from this analysis were entirely consistent with those from the main analysis. For example, all-cause inpatient visits (RR 1.86; 95% CI 1.81–1.91), all-cause ER visits (RR 1.09; 95% CI 1.06–1.11), CV-related outpatient visits (RR 2.32; 95% CI 2.28–2.35), and CV-related ER visits (RR 1.41; 95% CI 1.34–1.47) were significantly higher among patients with AFL (ie, those identified based on having 1 visit with a diagnosis of AFL) compared to patients without AFL. Fifth, not all potential confounders were available in this dataset, and unadjusted confounders may bias our findings. Lastly, our study did not take into consideration indirect medical (eg, costs associated with traveling to medical appointments) or nonmedical (eg, loss of productivity) expenses associated with AFL.

## Conclusion

Our results highlight the significant health care impact of AFL with or without concomitant AF. The incremental burden imposed by AFL was consistent across analysis by sex, AFL type, and with the removal of patients with concomitant AF, thereby highlighting the robustness of these results. Our results could serve to guide and support policymakers in resource allocation decisions. Furthermore, our results emphasize indicate the importance of treating and managing AFL using effective treatment strategies such as catheter ablation.
