# Supplementary material for: Atrial flutter-related health care use and costs: An analysis of a nationally representative administrative claims database in the United States
Source: Heart Rhythm O2. 2023 Apr 24;4(6):367–73. doi: 10.1016/j.hroo.2023.04.003 (PMC10288018; doi:10.1016/j.hroo.2023.04.003)
Supplement: Supplementary Data [file mmc1.docx]

**Supplemental Table 1. Health care use in the 12-month post-index diagnosis period among patients with incident atrial flutter (AFL) without concomitant atrial fibrillation (AF)**

| **Outcome** | **AFL**  **N = 5,150** | **No AFL**  **N = 5,159** | **p-value*** | **Relative Risk** | **95% Confidence Interval** |
| --- | --- | --- | --- | --- | --- |
| **All-cause health care use** | | | | | |
| Inpatient visits | 48.6% | 24.4% | < 0.001 | 2.48 | 2.38-2.60 |
| Outpatient visits | 99.6% | 97.7% | < 0.001 | 1.02 | 1.01-1.02 |
| ER visits | 38.3% | 30.7 | < 0.001 | 1.31 | 1.25-1.37 |
| Other medical visits^ | 67.3% | 60.1% | < 0.001 | 1.18 | 1.15-1.22 |
| **CV-related health care use** | | | | | |
| Inpatient visits | 41.5% | 12.1% | < 0.001 | 4.60 | 4.36-4.85 |
| Outpatient visits | 97.9% | 57.8% | < 0.001 | 3.30 | 3.25-3.34 |
| ER visits | 15.1% | 5.7% | - | - | - |
| Other medical visits^ | 36.0% | 21.1% | < 0.001 | 1.93 | 1.83-2.03 |
| Data are n (%).  *From bivariate regression model.  ^Other medical visits included any medical visits that were not captured with the inpatient, outpatient or ER visit categories (e.g., pharmacy, ambulance, residential substance abuse treatment facility).  AFL, atrial flutter; CI, confidence interval; CV, cardiovascular; ER, emergency room | | | | | |

**Supplemental Table 2. Average health care costs per patient in the 12-month post-index diagnosis period among patients with incident atrial flutter (AFL) without concomitant atrial fibrillation (AF)**

|  | **AFL**  **mean cost (SD)** | **No AFL**  **mean cost (SD)** | **Mean cost difference**  **(95% CI)** | **p-value*** |
| --- | --- | --- | --- | --- |
| **All-cause health care cost** |  |  |  |  |
| Inpatient visits | $27,318 ($80,218) | $14,032 ($57,442) | $13,286 ($11,031-$15,540) | < 0.001 |
| Outpatient visits | $23,057 ($38,172) | $10,345 ($25,877) | $12,712 ($11,648-$13,776) | < 0.001 |
| ER visits | $5,424 ($30,927) | $3,474 ($14,477) | $1,950 ($1,095-$2,805) | < 0.001 |
| Other medical visits^ | $3,346 ($28,031) | $2,160 ($17,585) | $1,186 ($405-$1,966) | 0.00291 |
| **CV-related health care costs** |  |  |  |  |
| Inpatient visits | $13,006 ($46,846) | $2,671 ($20,315) | $10,335 ($9,043-$11,628) | < 0.001 |
| Outpatient visits | $14,237 ($30,781) | $1,099 ($5,645) | $13,138 ($12,296-$13,981) | < 0.001 |
| ER visits | $1,018 ($12,213) | $141 ($2,219) | $877 ($543-$1,211) | < 0.001 |
| Other medical visits^ | $539 ($3,199) | $261 ($2,054) | $278 ($189-$368) | < 0.001 |
| **Prescription costs** | $5,782 ($13,759) | $3,731 ($12,957) | $2,051 ($1,662-$2,441) | < 0.001 |
| **Total health care costs** | $64,928 ($106,102) | $33,742 ($78,152) | $31,186 ($28,204-$34,166) | < 0.001 |
| Data are mean United States dollars (SD).  *From bivariate regression model.  ^Other medical visits included any medical visits that were not captured with the inpatient, outpatient or ER visit categories (e.g., pharmacy, ambulance, residential substance abuse treatment facility).  AFL, atrial flutter; CI, confidence interval; CV, cardiovascular; ER, emergency room; SD, standard deviation | | | | |
